# Supplementary material for: Store-Operated Calcium Entry Increases Nuclear Calcium in Adult Rat Atrial and Ventricular Cardiomyocytes
Source: Cells. 2023 Nov 23;12(23):2690. doi: 10.3390/cells12232690 (PMC10705675; doi:10.3390/cells12232690)

Supplementary Material

Store-operated calcium entry increases nuclear calcium in adult rat atrial and ventricular cardiomyocytes

Julia Hermes[1], Vesela Borisova[1,2] and Jens Kockskämper[1]

[1] Institute for Pharmacology and Clinical Pharmacy, Biochemical and Pharmacological Centre (BPC) Marburg, University of Marburg, Karl-von-Frisch-Str. 2 K|03, 35043 Marburg, Germany

[2] Department of Pharmacology and Clinical Pharmacology and Therapeutics, Medical University of Varna, Prof. dr. Marin Drinov str. 55, Varna, Bulgaria

Corresponding author:
Prof. Dr. Jens Kockskämper
jens.kockskaemper@staff.uni-marburg.de

Supplementary Figures

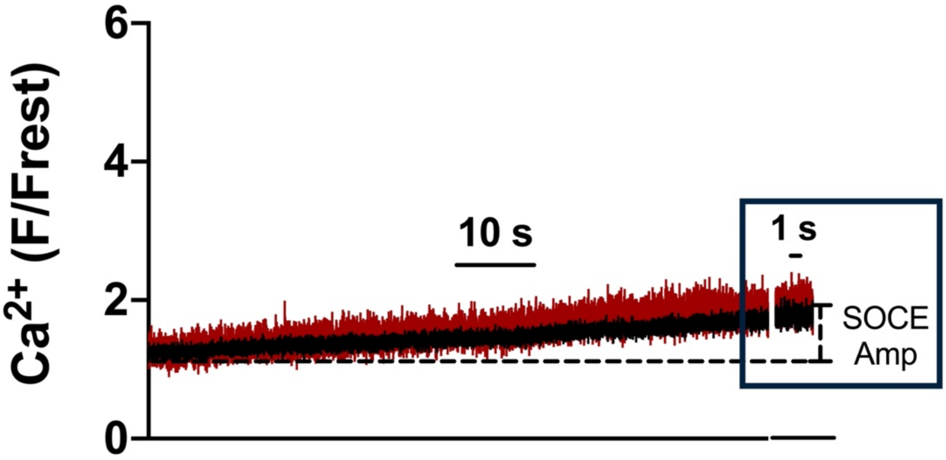

Supplementary Figure S1 Determination of SOCE amplitudes. Traces show cytosolic (black) and nuclear (red) fluorescence (Ca) increases in a ventricular myocyte following re-addition of 2 mM Ca solution after complete store depletion (trace (j) in Figure 1). A few seconds after the 90 s recording (represented by the gap), another 5 s recording was conducted to determine SOCE amplitudes, as indicated (SOCE Amp). For simplicity, the final 5 s recording has been omitted from the respective figures, in which SOCE traces are shown (Figures 3-7).

Supplementary Figure S2 Original immunoblot images underlying the data presented in Figure 8

TRPC1 (Alternating four ventricular and four atrial samples from WKY, from four different animals. Membranes were cut before antibody application. Upper membrane contains samples #1-4 of each tissue type, lower membrane samples #5-8 of each tissue type. Upper membrane (cropped) is presented in Figure 8.)

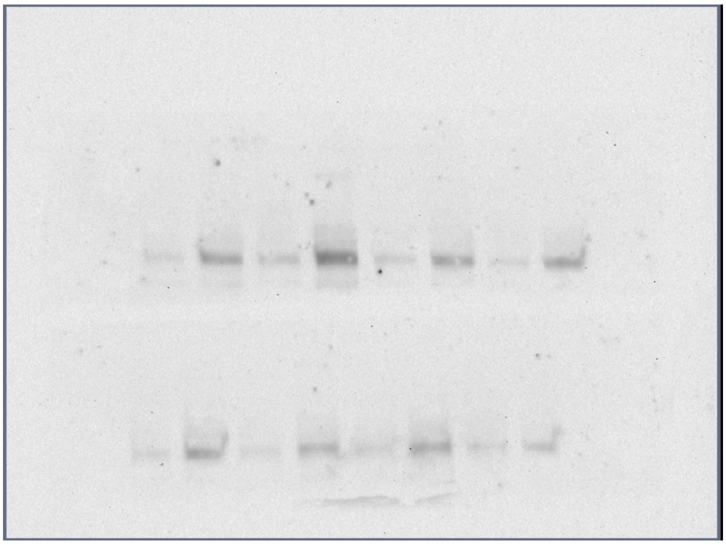

Associated GAPDH membranes (Upper membrane contains samples #1-4 of each tissue type, lower membrane samples #5-8 of each tissue type. Upper membrane (cropped) is presented in Figure 8.)

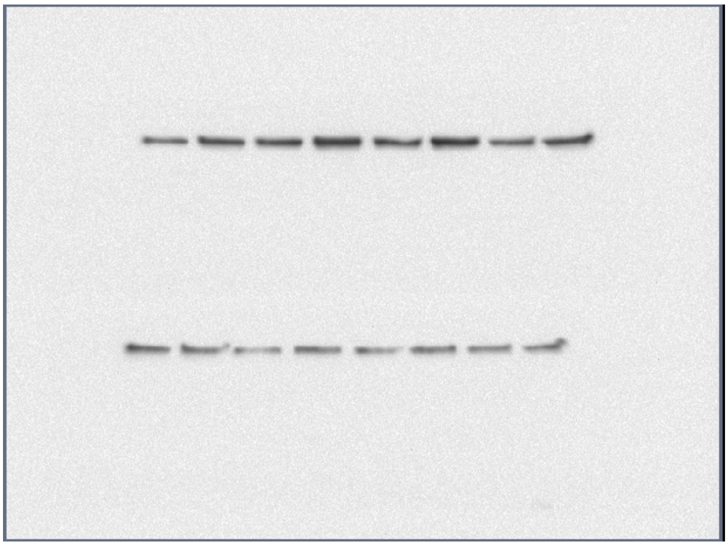

TRPC3 (Alternating four ventricular and four atrial samples from WKY, from four different animals. Membranes were cut before antibody application. Upper membrane contains samples #1-4 of each tissue type, lower membrane samples #5-8 of each tissue type. Lower membrane (cropped) is presented in Figure 8.)

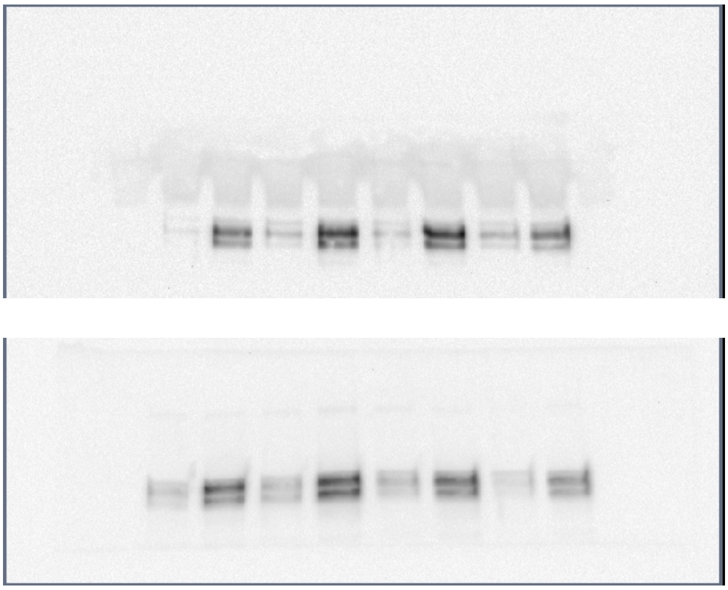

Associated GAPDH membranes (Upper membrane contains samples #1-4 of each tissue type, lower membrane samples #5-8 of each tissue type. Lower membrane (cropped) is presented in Figure 8.)

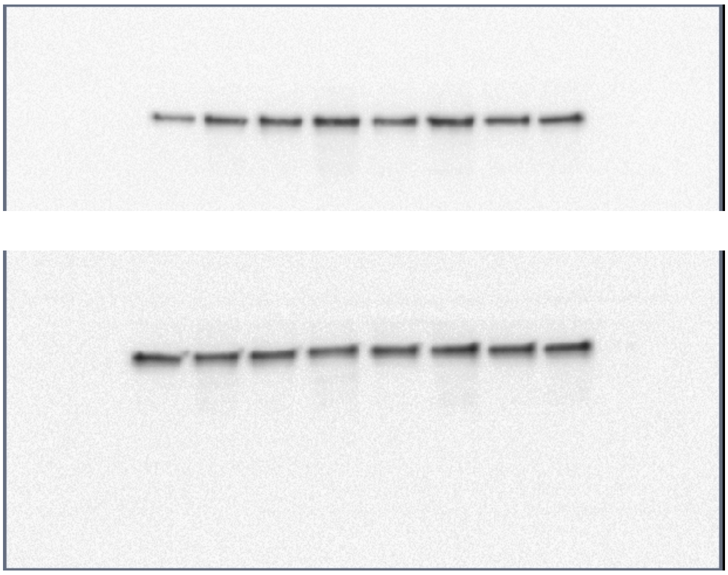

TRPC6 (Alternating four ventricular and four atrial samples from WKY, from four different animals. Membranes were cut before antibody application. Upper membrane contains samples #1-4 of each tissue type, lower membrane samples #5-8 of each tissue type. Upper membrane (cropped) is presented in Figure 8.)

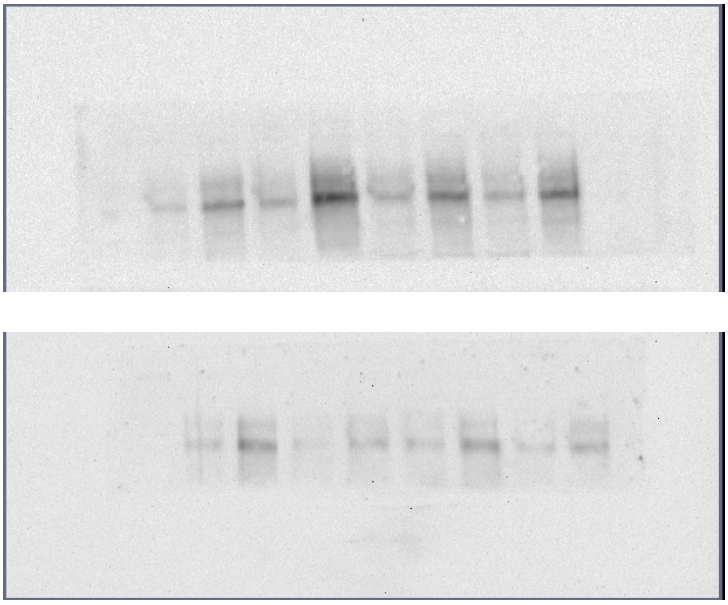

Associated GAPDH membranes (Upper membrane contains samples #1-4 of each cell type, lower membrane samples #5-8 of each cell type. Upper membrane (cropped) is presented in Figure 8.)

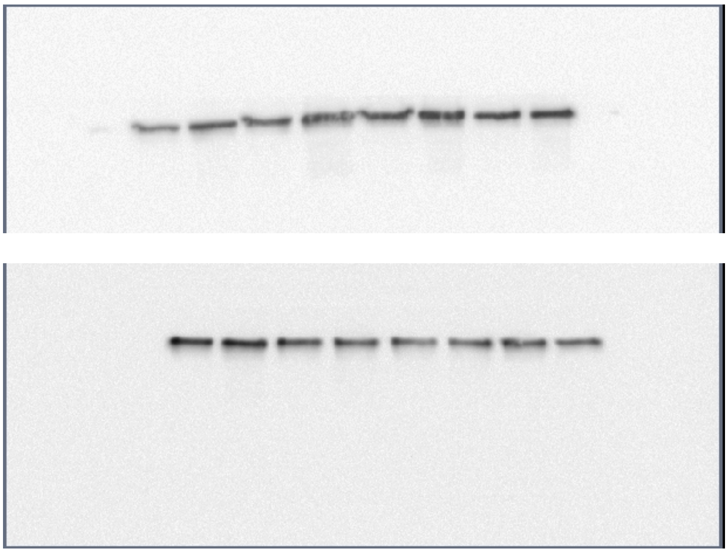

STIM1 (Alternating four ventricular and four atrial samples from WKY, from four different animals. Membranes were cut before antibody application. Upper membrane contains samples #1-4 of each tissue type, lower membrane samples #5-8 of each tissue type. Upper membrane (cropped) is presented in Figure 8.)

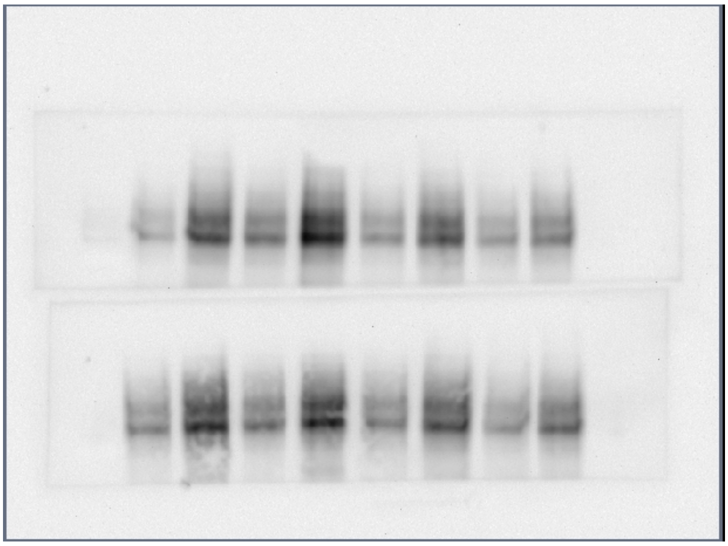

Associated GAPDH membranes (Upper membrane contains samples #1-4 of each tissue type, lower membrane samples #5-8 of each tissue type. Upper membrane (cropped) is presented in Figure 8.)

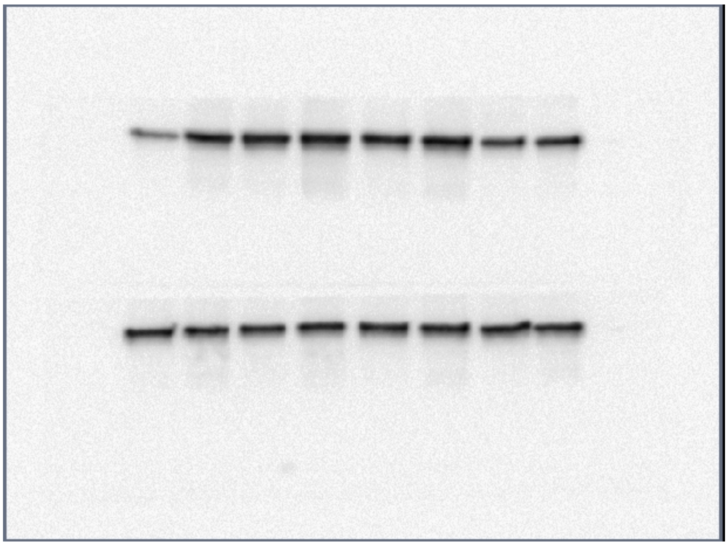

Representative membranes with molecular weight markers (bands at the left) after cutting slightly below 55 kDa, which were always loaded on the left well of each gel. Left hand side shows upper parts of the membranes with molecular weight markers of 250 kDa, 130 kDa, 100 kDa, 70 kDa and 55 kDa; right hand side shows lower parts of the membranes with molecular weight markers of 35 kDa, 25 kDa and 15 kDa.

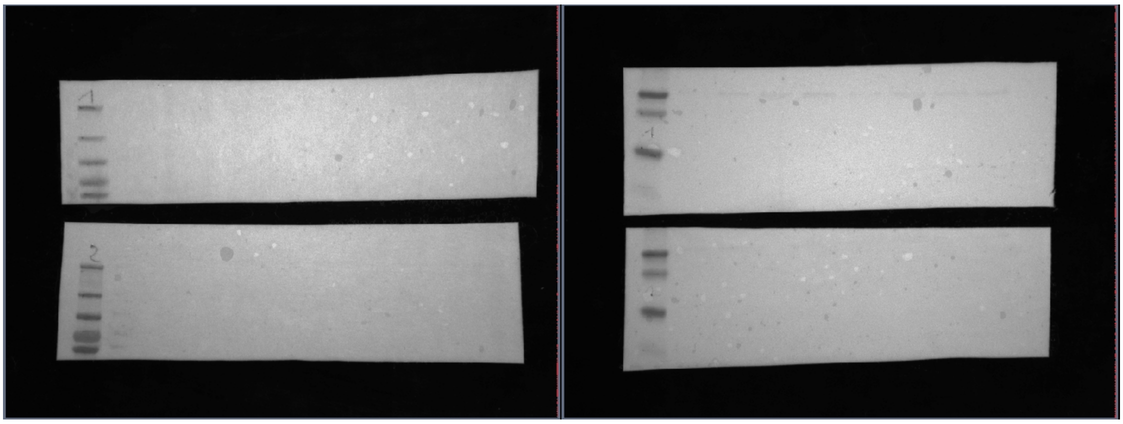

Supplement: Supplementary file 1 [file cells-12-02690-s001.zip › cells-2652958-supplementary.pdf]
